# Data on fatty acid profiles of oils isolated from the mesocarps and seeds of four avocado species derived from Sabah, Malaysia

**DOI:** 10.1016/j.dib.2025.112259

**Published:** 2025-11-12

**Authors:** Nurul Ashikin Nasir, Peik Lin Teoh, Nurul Elyani Mohamad, Duane Evans, Md. Shafiquzzaman Siddiquee, Bo Eng Cheong

**Affiliations:** a Biotechnology Research Institute, Universiti Malaysia Sabah, Jalan UMS, 88400 Kota Kinabalu, Sabah, Malaysia; b Sabah Durian and Tropical Fruit Planters Association, 2nd Floor, Lot 30, Block C, Damai Point, 88300 Kota Kinabalu, Sabah, Malaysia

**Keywords:** Persea americana, QAV1, QAV2, Short neck russell, Local bacon, Oil composition, GC-FID

## Abstract

This dataset presents detailed fatty acid composition data from the mesocarp and seed of four avocado varieties cultivated in Sabah, Malaysia. Avocado is a sought-after superfood, largely due to its high content of health-benefiting unsaturated fatty acids. While varieties like Hass and Fuerte have been well studied, no reports exist on the lipid profiles of avocados grown in Sabah—a region increasingly active in avocado farming. This study addresses that gap by analyzing the fatty acid profiles of four Sabah-derived varieties: Quoin Avocado 1 (QAV1), Quoin Avocado 2 (QAV2), Short Neck Russell, and Local Bacon, with Australian Hass as a reference. A total of 45 mesocarp and 25 seed samples were collected from fruits grown in the Keningau area. The samples were freeze-dried, and total fats were extracted following AOAC guidelines. The extracted lipids were converted to fatty acid methyl esters (FAMEs) and analyzed using Gas Chromatography-Flame Ionization Detection (GC-FID). The fatty acids were classified into saturated (SFA), monounsaturated (MUFA), and polyunsaturated (PUFA) categories for comparative evaluation. The dataset includes a comprehensive profile of 37 FAMEs, enabling in-depth comparisons between the local varieties and the commercial Hass avocado. The findings offer valuable insights into the nutritional value, genetic diversity, and potential commercial applications of Sabah avocados, contributing to future avocado breeding efforts and regional agronomic development.

Specifications Table

**Table:** 

Subject	Biochemistry
Specific subject area	Plant Biochemistry
Data format	Raw, Analyzed
Type of data	Table, Figure
Data collection	Fruits of the four avocado varieties derived from Sabah of Malaysia were collected from orchards located at Keningau district of Sabah from September 2022 until November 2022. Fruits of the Hass variety (derived from Australia) were purchased from local supermarkets. All the fruits were processed after they were fully ripe, in which the mesocarp and seed parts were collected. The extraction of oil from the mesocarp and seed samples was conducted by referring to AOAC 20th Edition, 991.36. The fatty acid methyl esters (FAMEs) were prepared by derivatizing the avocado mesocarp and seed oil samples using boron trifluoride-methanol solution. The FAME clear solution was separated out from the cloudy aqueous layer. For GC-FID analysis, an Agilent (Santa Clara, CA, USA) 7890 GC-FID system equipped with an Agilent 7693 autosampler was used. Calibration mixture was prepared from a Supelco 37 component FAME mixture (Sigma-Aldrich, St. Louis, Missouri, United States) with methyl nonadecanoate used as internal standard (ISTD). The 37 FAME mixture consisted of C4:0, C6:0, C8:0, C10:0, C11:0, C12:0, C13:0, C14:0, C15:0, C16:0, C17:0, C18:0, C20:0, C21:0, C22:0, C23:0, C24:0, C14:1, C15:1, C16:1, C17:1, C18:1n9t, C18:1n9c, C20:1n9, C22:1n9, C24:1, C18:2n6t, C18:2n6c, C18:3n6, C18:3n3, C20:2, C20:3n6, C30:3n3, C20:4n6, C20:5n3, C22:2, C22:6n3.
Data source location	Institution: Biotechnology Research Institute, Universiti Malaysia Sabah, Kota Kinabalu, Malaysia. City: Kota Kinabalu Country: Malaysia
Data accessibility	Repository name: Mendeley data Data identification number: DOI: 10.17632/2j3sk5tt53.2 Direct URL to data: https://data.mendeley.com/datasets/2j3sk5tt53/2

## 1 Value of the Data

• This article presents detailed data on the fatty acid composition of four avocado varieties widely cultivated and derived from Sabah, Malaysia—namely Quoin Avocado 1 (QAV1), Quoin Avocado 2 (QAV2), Short Neck Russell (SNR), and Local Bacon (LB)—analyzing both mesocarp and seed components.
• These data mark an important step in establishing a baseline for the fatty acid profiles of Sabah’s avocado varieties, contributing valuable insights into their nutritional characteristics - an area that remains largely underexplored.
• The dataset generated offers significant potential benefits for both local growers and consumers. For growers, it provides essential nutritional information that can be used to market Sabah avocados as rich sources of health-promoting fatty acids, appealing to health-conscious consumers. For the public, increased awareness of these nutritional benefits may encourage greater avocado consumption as part of a healthy diet.
• As the first comprehensive profiling of these locally cultivated varieties, the data serves as a foundational reference for future research and development. It can support the formulation of new avocado-based products, as well as improvements in agricultural practices tailored to optimize both yield and nutritional quality.

## 2 Background

Avocado is a highly sought-after superfood known for its health-promoting unsaturated fatty acids. While extensive fatty acid analyses have been conducted on well-known varieties such as Hass and Fuerte, no studies have examined Sabah’s avocados, despite the state’s active avocado farming industry. Sabah is one of Malaysia’s most agriculturally rich states, characterized by a humid and tropical climate. Over the years, avocado farming in Sabah has expanded steadily, supported by the state government, which has allocated approximately 3237 hectares of land for avocado plantations [1]. Most of this cultivated area is concentrated in the Keningau, Tenom, Tawau, Lahad Datu, Tuaran, Ranau and Beaufort districts, where environmental conditions favor fruit development. According to the 2023 State Annual Report, Sabah’s avocado production reached 323.7 tons that year and has shown a consistent upward trend [2]. This research examines the fatty acid profiles of four Sabah avocado varieties—Quoin Avocado 1 (QAV1), Quoin Avocado 2 (QAV2), Short Neck Russell (SNR) and Local Bacon (LB), which the results will be compared to the commercial Hass variety. QAV1 is a large, pear-shaped variety that turns red-purplish when ripe, while QAV2 is smaller and oval-shaped, with green skin that develops dark spots as it ripens. SNR variety, the largest among the four, featuring a bottle-necked shape and also turns red-purplish when ripe. Local Bacon, is small, elongated and pear-shaped, with green skin that develops dark spots upon ripening. Both QAV1 and QAV2 varieties were bred by the Quoin Hill Agriculture Research Centre and officially introduced in 2012, whereas the other two were bred by the local planters [3]. The four avocado varieties exhibit a biannual fruiting pattern, with the first harvest period occurring between March and June, and the second between September and October. Overall, the dataset from this study provides valuable information for future breeding programs, nutritional research and the commercial applications of these local avocado varieties.

## 3 Data Description

**Fig. 1:**
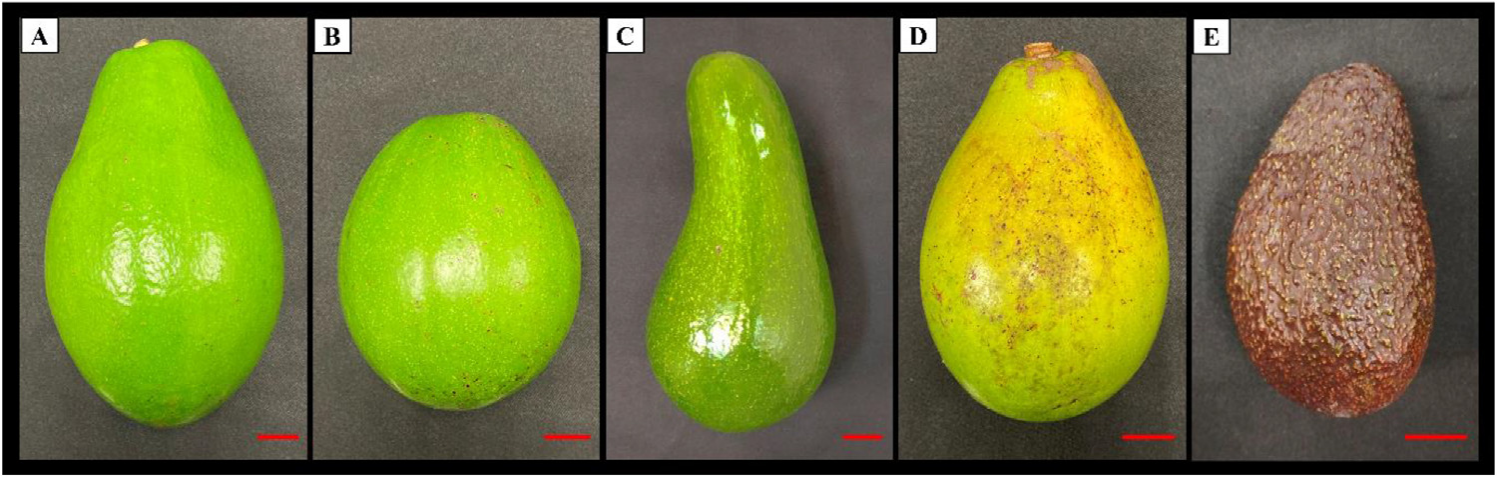
Photograph of Sabah’s avocado varieties and Hass variety. [A] Quoin avocado 1 (QAV1). [B] Quoin avocado 2 (QAV2). [C] Short Neck Russell. [D] Local Bacon. [E] Hass. Scale bar = 2 cm.

**Fig. 2:**
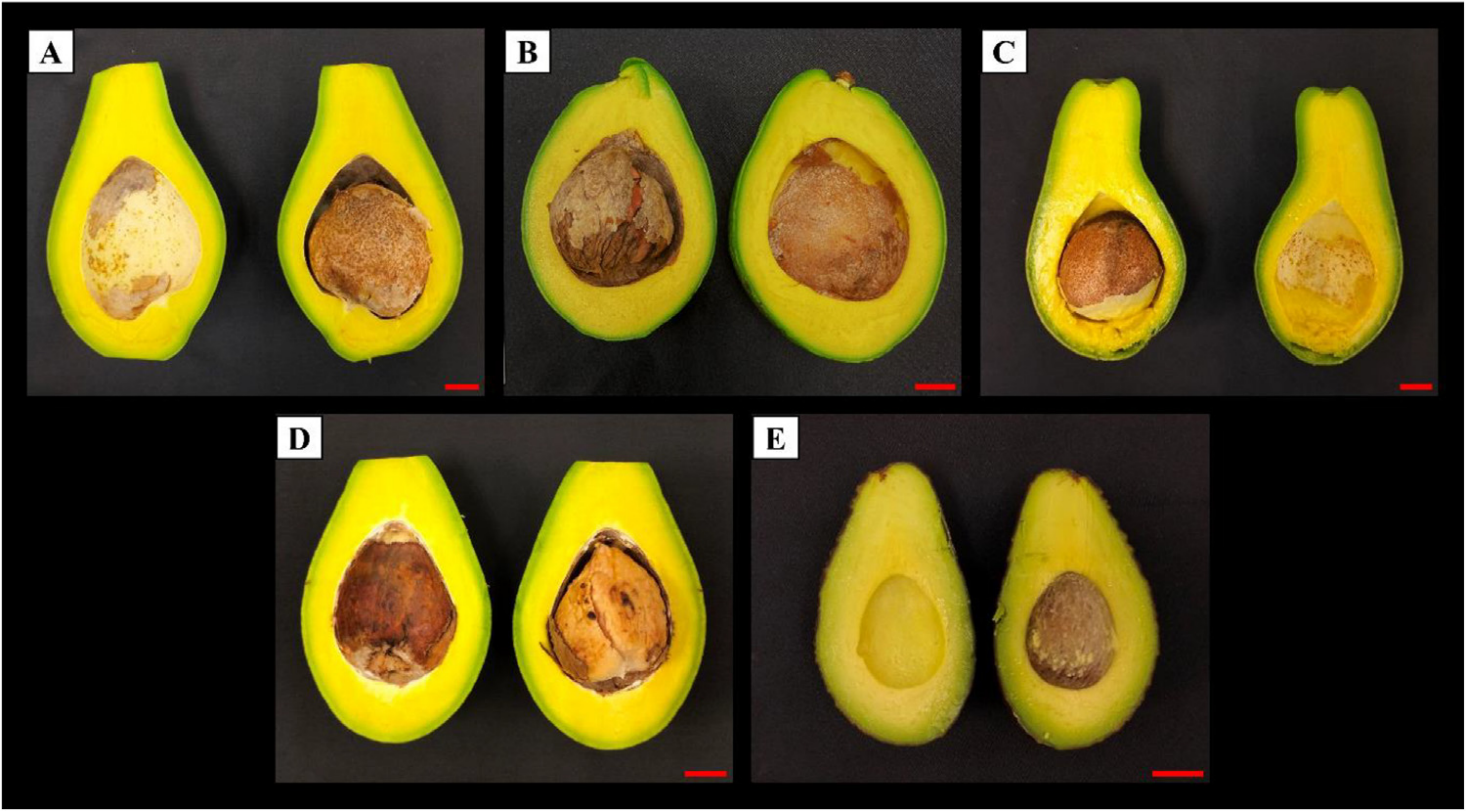
Photograph of cross-section of Sabah’s avocado varieties and Hass variety. [A] Quoin avocado 1 (QAV1). [B] Quoin avocado 2 (QAV2). [C] Short Neck Russell. [D] Local Bacon. [E] Hass. Scale bar = 2 cm.

This article presents the dataset detailing the fatty acid compositions in the mesocarps and seeds of four commonly grown avocado varieties derived from Sabah, Malaysia. The four varieties are Quoin Avocado 1 (QAV1), Quoin Avocado 2 (QAV2), Short Neck Russell, and Local Bacon. Besides, the Hass variety was used as control for comparison purpose. Fig. 1 provides a visual representation of these four Sabah avocado varieties; Fig. 2 shows the cross sections of the avocado varieties used in this study. The experimental findings of the fatty acid compositions in the mesocarps of the four Sabah avocado varieties and the Hass variety are presented in Table 1. Additionally, data on fatty acid profiles of the seeds are provided in Table 2.

**Table 1:** Composition of fatty acids in the mesocarps of four Sabah avocado varieties and the Hass variety.

Fatty acid ( %)	Hass (*n* = 5)	QAV1 (*n* = 10)	QAV2 (*n* = 10)	Short Neck Russell (*n* = 10)	Local Bacon (*n* = 10)
Saturated fatty acids					
C8:0	0.04 ± 0.08	0.01 ± 0.01	0.27 ± 0.83	0.01 ± 0.01	0.01 ± 0.02
C10:0	0.03 ± 0.04	0.01 ± 0.01	0.14 ± 0.43	0.01 ± 0.00	0.01 ± 0.01
C12:0	0.01 ± 0.00	0.09 ± 0.06	0.08 ± 0.04	0.08 ± 0.05	0.02 ± 0.00
C14:0	0.06 ± 0.01	0.12 ± 0.04	0.11 ± 0.02	0.13 ± 0.02	0.14 ± 0.03
C15:0	0.04 ± 0.01	0.04 ± 0.01	0.04 ± 0.01	0.04 ± 0.01	0.06 ± 0.01
C16:0	22.91 ± 1.91	30.92 ± 1.13	33.44 ± 0.76	29.04 ± 0.69	26.28 ± 1.12
C17:0	0.02 ± 0.00	0.04 ± 0.00	0.03 ± 0.00	0.03 ± 0.01	0.04 ± 0.01
C18:0	0.54 ± 0.03	0.94 ± 0.12	0.68 ± 0.04	0.94 ± 0.06	1.08 ± 0.05
C20:0	0.07 ± 0.01	0.17 ± 0.03	0.10 ± 0.01	0.15 ± 0.01	0.17 ± 0.01
C21:0	0.00 ± 0.00	0.04 ± 0.04	0.04 ± 0.03	0.08 ± 0.05	0.12 ± 0.06
C22:0	0.04 ± 0.02	0.05 ± 0.01	0.06 ± 0.06	0.04 ± 0.03	0.05 ± 0.05
C24:0	0.34 ± 0.09	0.06 ± 0.03	0.13 ± 0.12	0.15 ± 0.16	0.14 ± 0.07
Total	24.10 ± 2.00	32.48 ± 1.09	35.12 ± 0.89	30.72 ± 0.72	28.13 ± 1.12
Monounsaturated fatty acids					
C14:1	0.01 ± 0.001	0.01 ± 0.00	0.01 ± 0.00	0.01 ± 0.00	0.01 ± 0.01
C15:1	0.02 ± 0.004	0.05 ± 0.01	0.02 ± 0.01	0.03 ± 0.01	0.03 ± 0.01
C16:1	11.80 ± 1.08	11.31 ± 1.12	17.63 ± 0.48	9.48 ± 0.80	9.09 ± 0.32
C17:1	0.08 ± 0.005	0.09 ± 0.01	0.10 ± 0.00	0.09 ± 0.01	0.09 ± 0.01
C18:1n9c	49.01 ± 1.97	37.23 ± 2.94	30.76 ± 2.10	44.56 ± 2.29	39.40 ± 2.35
C20:1n9	0.19 ± 0.01	0.19 ± 0.02	0.17 ± 0.01	0.16 ± 0.01	0.14 ± 0.01
C24:1	0.00 ± 0.00	0.18 ± 0.10	0.00 ± 0.00	0.01 ± 0.05	0.05 ± 0.09
Total	61.11 ± 1.02	49.06 ± 2.19	48.68 ± 2.11	54.33 ± 1.77	48.81 ± 2.30
Polyunsaturated fatty acids					
C18:2n6c	14.08 ± 1.33	16.76 ± 1.46	14.66 ± 1.68	13.63 ± 1.12	20.33 ± 1.31
C18:3n6	0.07 ± 0.03	0.06 ± 0.17	0.02 ± 0.02	0.01 ± 0.03	0.03 ± 0.02
C18:3n3	0.65 ± 0.11	1.63 ± 0.34	1.51 ± 0.28	1.30 ± 0.28	2.69 ± 0.32
Total	14.80 ± 1.44	18.46 ± 1.81	16.20 ± 1.96	14.95 ± 1.38	23.06 ± 1.61

The results are presented in mean ± standard deviation. Only fatty acid detected and quantified was shown in the table. C8:0, caprylic acid; C10:0, capric acid; C12:0, lauric acid; C14:0, myristic acid; C15:0, pentadecanoic acid; C16:0, palmitic acid; C17:0, heptadecanoic acid; C18:0, stearic acid; C20:0, arachidic acid; C21:0, henicosanoic acid; C22:0, behenic acid; C24:0, lignoceric acid; C14:1, myristoleic acid; C15:1, cis-10-pentadecenoic; C16:1, palmitoleic acid, C17:1, cis-10-heptadecanoic; C18:1n9c, oleic acid; C20:1n9, cis-11-eicosenoic; C24:1, nervonic; C18:2n6c, linoleic (*cis*); C18:3n6, γ-linolenic; C18:3n3, α-linolenic.

**Table 2:** Composition of fatty acids in the seeds of four Sabah avocado varieties and the Hass variety.

Fatty acid ( %)	Hass	QAV1	QAV2	Short Neck Russell	Local Bacon
Saturated fatty acids					
C6:0	0.02 ± 0.01	0.03 ± 0.01	0.03 ± 0.02	0.03 ± 0.01	0.10 ± 0.04
C8:0	0.14 ± 0.13	0.27 ± 0.21	0.14 ± 0.05	0.16 ± 0.06	0.99 ± 0.44
C10:0	0.12 ± 0.11	0.23 ± 0.19	0.11 ± 0.05	0.15 ± 0.06	0.89 ± 0.40
C12:0	1.07 ± 0.90	2.27 ± 1.74	1.10 ± 0.49	1.51 ± 0.58	7.63 ± 3.12
C13:0	0.03 ± 0.01	0.03 ± 0.01	0.04 ± 0.01	0.02 ± 0.02	0.02 ± 0.01
C14:0	1.39 ± 0.53	2.92 ± 1.09	2.57 ± 0.66	1.95 ± 0.39	5.15 ± 1.21
C15:0	0.77 ± 0.62	1.50 ± 1.45	3.08 ± 0.64	0.69 ± 0.43	0.39 ± 0.07
C16:0	19.92 ± 6.94	26.59 ± 1.17	26.62 ± 3.72	28.36 ± 3.01	30.64 ± 3.42
C17:0	0.19 ± 0.06	0.37 ± 0.07	0.37 ± 0.10	0.28 ± 0.09	0.38 ± 0.08
C18:0	2.05 ± 0.91	6.41 ± 2.97	4.44 ± 1.23	6.53 ± 5.30	4.87 ± 1.23
C20:0	0.37 ± 0.10	0.65 ± 0.11	0.71 ± 0.23	0.64 ± 0.15	0.49 ± 0.15
C21:0	0.01 ± 0.01	0.02 ± 0.04	0.08 ± 0.08	0.01 ± 0.03	0.00 ± 0.00
C22:0	0.33 ± 0.16	0.51 ± 0.08	0.59 ± 0.22	0.59 ± 0.16	0.46 ± 0.10
C23:0	0.14 ± 0.03	0.32 ± 0.05	0.39 ± 0.05	0.34 ± 0.08	0.32 ± 0.06
C24:0	0.50 ± 0.22	1.19 ± 0.82	1.80 ± 0.57	0.98 ± 0.26	0.62 ± 0.16
Total	27.05 ± 7.79	43.30 ± 3.37	42.07 ± 4.89	42.24 ± 3.48	52.96 ± 7.91
Monounsaturated fatty acids					
C14:1	4.52 ± 1.43	5.14 ± 2.94	7.07 ± 2.23	4.51 ± 5.06	1.87 ± 0.98
C15:1	0.23 ± 0.12	0.16 ± 0.13	0.20 ± 0.10	0.14 ± 0.10	0.06 ± 0.04
C16:1	6.93 ± 3.14	3.43 ± 1.77	3.97 ± 1.39	4.71 ± 2.11	4.45 ± 1.76
C17:1	0.59 ± 0.07	0.51 ± 0.24	0.51 ± 0.26	0.44 ± 0.27	0.34 ± 0.20
C18:1n9t	0.13 ± 0.10	0.06 ± 0.09	0.11 ± 0.07	0.10 ± 0.11	0.25 ± 0.45
C18:1n9c	22.89 ± 10.16	16.59 ± 3.28	12.33 ± 1.83	14.68 ± 7.47	11.38 ± 8.76
C20:1n9	0.57 ± 0.55	0.34 ± 0.12	0.38 ± 0.18	0.25 ± 0.09	0.31 ± 0.29
C22:1n9	0.19 ± 0.25	0.05 ± 0.09	0.05 ± 0.06	0.06 ± 0.11	0.18 ± 0.25
C24:1	0.54 ± 0.52	0.68 ± 0.36	0.76 ± 0.35	0.44 ± 0.24	0.23 ± 0.06
Total	36.60 ± 11.48	26.96 ± 2.23	25.38 ± 3.75	25.33 ± 9.05	19.08 ± 7.75
Polyunsaturated fatty acids					
C18:2n6t	0.25 ± 0.13	0.25 ± 0.19	0.22 ± 0.08	0.18 ± 0.10	0.09 ± 0.09
C18:2n6c	18.28 ± 4.39	24.16 ± 4.58	25.56 ± 3.09	27.23 ± 5.90	23.21 ± 6.05
C18:3n6	5.17 ± 5.34	0.20 ± 0.09	2.33 ± 1.20	0.26 ± 0.33	0.74 ± 0.34
C18:3n3	2.54 ± 0.47	3.51 ± 0.71	3.27 ± 0.93	3.84 ± 1.23	2.65 ± 0.96
C20:2	9.13 ± 9.93	0.20 ± 0.14	0.12 ± 0.05	0.19 ± 0.13	0.29 ± 0.13
C20:3n3	0.17 ± 0.19	0.06 ± 0.07	0.11 ± 0.04	0.25 ± 0.14	0.47 ± 0.10
C20:4n6	0.08 ± 0.11	0.02 ± 0.05	0.03 ± 0.06	0.00 ± 0.00	0.00 ± 0.00
C20:5n3	0.15 ± 0.05	0.46 ± 0.47	0.21 ± 0.05	0.12 ± 0.04	0.13 ± 0.09
C22:2	0.41 ± 0.27	0.02 ± 0.04	0.18 ± 0.06	0.10 ± 0.08	0.08 ± 0.10
C22:6n3	0.16 ± 0.17	0.85 ± 0.38	0.52 ± 0.35	0.27 ± 0.42	0.32 ± 0.32
Total	36.35 ± 18.84	29.73 ± 5.28	32.55 ± 3.97	32.43 ± 7.57	27.97 ± 7.14

The results are presented in mean ± standard deviation. (*n* = 5). Only fatty acid detected and quantified was shown in the table. C6:0, caproic acid; C8:0, caprylic acid; C10:0, capric acid; C12:0, lauric acid; C13:0, tridecanoic acid; C14:0, myristic acid; C15:0, pentadecanoic acid; C16:0, palmitic acid; C17:0, heptadecanoic acid; C18:0, stearic acid; C20:0, arachidic acid; C21:0, henicosanoic acid; C22:0, behenic acid; C23:0, tricosanoic acid; C24:0, lignoceric acid; C14:1, myristoleic acid; C15:1, cis-10-pentadecenoic; C16:1, palmitoleic acid, C17:1, cis-10-heptadecanoic; C18:1n9t, elaidic (trans); C18:1n9c, oleic acid; C20:1n9, cis-11-eicosenoic; C22:1n9, erucic acid; C24:1, nervonic; C18:2n6t, linolelaidic acid (trans); C18:2n6c, linoleic (*cis*); C18:3n6, γ-linolenic; C18:3n3, α-linolenic; C20:2, cis-11,14-eicosadienoic acid; C20:3n6, cis-8,11,14-eicosatrienoic acid; C20:3n3, cis-11,14,17-eicosatrienoic acid; C20:4n6, arachidonic acid; C20:5n3, cis-5,8,11,14,17-eicosapentaenoic acid; C22:2, cis-13,16-docosadienoic acid; C22:6n3, cis-4,7,10,13,16,19-docosahexaenoic acid.

## 4 Experimental Design, Materials and Methods

### 4.1 Plant materials

Between September and November of 2022, a total of 60 avocado varieties native to Sabah were harvested from an orchard owned by a member of the Sabah Durian and Tropical Fruit Planters Association (MasDA) located in Keningau. Additionally, ten Hass avocados imported from Australia were bought from a local supermarket. Upon collection, the fruits underwent a thorough washing with tap water, followed by a rinse with distilled water, and gentle drying with a kitchen towel. Following this cleaning process, the fruits were placed in an environment at room temperature to facilitate ripening. Once fully ripe, the avocados were carefully cut open, and their mesocarps and seeds were prepared for further analysis. Both mesocarps and seeds were subjected to a pre-treatment, involving freeze-drying using Labconco (Kansas City, Missouri, USA) Model 12 Port Dry Ice Benchtop Freeze Dry System, spanned over a period of seven days. Subsequently, the freeze-dried mesocarps and seeds were meticulously pulverized into a fine powder utilizing a stainless-steel blender. The resulting powder was then vacuum sealed and stored at a temperature of −20 °C prior fatty acid analysis and oil extraction.

### 4.2 Fatty acid profiling using gas chromatography-flame ionization detection (GC-FID)

For the fatty acid profiling of avocado mesocarp, a total of 45 mesocarp samples, with ten samples from each variety weighing 15±0.5 g, were prepared. Similarly, for the seed samples, 25 samples were prepared, with five samples for each variety, each weighing 15±0.5 g. All samples were sent to the National University of Malaysia (UKM) UNIPEQ for a thorough fatty acid analysis. In brief, total fat extraction was conducted using a Soxhlet apparatus, following an in-house procedure designated as No: STP/Chem/A02, which is based on the AOAC 20th Edition: 991.36 [4]. Briefly, the sample powder was mixed with concentrated hydrochloric acid in methanol and heated in a water bath for 15 to 25 min. The resulting mixture was then underwent Soxhlet extraction using diethyl ether to obtain the fat content. The diethyl ether and extracted fat mixture was subsequently separated under reduced pressure using a rotary evaporator*.* Then, the transesterification of fatty acids to methyl esters and profiling procedures were adhered to the guidelines outlined in the AOAC 20th edition, specifically method 996.06, utilizing Gas Chromatography with Flame Ionization Detection (GC-FID) [5]. The extracted avocado oil underwent transesterification with boron trifluoride-methanol (BF_3_−MeOH) reagent to produce fatty acid methyl esters (FAME). The FAME clear solution was separated out from the cloudy aqueous layer. These FAMEs were analyzed using an Agilent (Santa Clara, CA, USA) GC-FID system equipped with an Agilent 7693 autosampler. A volume of one µL of FAME was precisely injected into the GC port. For separation, an Agilent J&W DB-225 (30 m, 0.25 mm, 0.20 µm) column and hydrogen gas (1.3 mL/min) mobile phase was used. The inlet temperature was 250 °C and the detector temperature was 260 °C. A split ratio of 50:1 was used. The temperature program started from 100 °C, which was kept constant for 3 min. Then, the column was heated at 20 °C/min to reach 166 °C, where it was kept for 5 min. Then, it was heated to 180 °C, at 1 °C/min, and finally to 240 °C at 10 °C/min, where it was kept for 1 min. Calibration mix was prepared from a Supelco 37 component FAME mixture (Sigma-Aldrich, St. Louis, Missouri, United States) with methyl nonadecanoate as internal standard (ISTD). The 37 FAMEs mixture consisted of C4:0, C6:0, C8:0, C10:0, C11:0, C12:0, C13:0, C14:0, C15:0, C16:0, C17:0, C18:0, C20:0, C21:0, C22:0, C23:0, C24:0, C14:1, C15:1, C16:1, C17:1, C18:1n9t, C18:1n9c, C20:1n9, C22:1n9, C24:1, C18:2n6t, C18:2n6c, C18:3n6, C18:3n3, C20:2, C20:3n6, C30:3n3, C20:4n6, C20:5n3, C22:2, C22:6n3.

## Limitations

None.

## Ethics Statement

This study does not involve human or animal experimental subjects, and the data collected were not gathered from social media platforms.

## CRediT Author Statement

**Nurul Ashikin Nasir:** Investigation, Data curation, Validation, Writing – Original Draft. **Peik Lin Teoh:** Validation, Supervision. **Nurul Elyani Mohamad:** Validation, Supervision. **Duane Evans:** Resources. **Md. Shafiquzzaman Siddiquee:** Validation, Supervision. **Bo Eng Cheong:** Conceptualization, Validation, Project administration, Writing – Review & Editing, Supervision.

## Data Availability

Mendeley DataData on fatty acid profiles of oils isolated from the mesocarps and seeds of four avocado species derived from Sabah, Malaysia (Original data).

## Appendix Supplementary materials

Image, application 1
